# Attentional cueing in numerical cognition

**DOI:** 10.3389/fpsyg.2014.01381

**Published:** 2014-12-01

**Authors:** Martin H. Fischer, André Knops

**Affiliations:** ^1^Division of Cognitive Science, University of PotsdamPotsdam - Golm, Germany; ^2^Emmy Noether Research Group Leader, Department of Psychology, Humboldt-University BerlinBerlin, Germany

**Keywords:** attention, number line, SNARC effect, visual perception, response selection

Fischer et al. (2003) discovered that non-informative central numerical cues induce attention shifts: In accordance with a spatially organized mental number line, small, and large numbers facilitated detection of targets in the left and right visual hemifields, respectively. Zanolie and Pecher (2014, henceforth Z&P) failed to observe this pattern in 5 of 6 replication attempts and concluded that “the mental number line is not activated automatically but at best only when it is contextually relevant.” We briefly describe the current understanding of spatial-numerical associations (SNAs) before identifying aspects of Z&P's study that might explain their failure to replicate the attentional cueing effect of numbers.

In addition to two successful replications acknowledged by Z&P (and a replication within the original paper), number-induced attentional biases were also found by several other labs (for references, see Fischer and Shaki, 2014, p. 1465). The effect has been extended to neuroscientific measures, such as modulations of neural activity in occipital cortex as measured with functional magnetic resonance imaging (Goffaux et al., 2014), or increased P1 and P3 amplitudes in EEG (Sallilas et al., 2008), and it is also reflected in attention-related ERP-components EDAN and ADAN (Ranzini et al., 2009) Recent evidence suggests that these ERP-correlates of number-induced attentional cueing are not restricted to mere detection tasks but generalize to color discrimination (Schuller et al., 2014). Moreover, number-induced attention shifts in EEG signatures even without behavioral bias, as in the Ranzini et al. (2009) study, can be interpreted as strong replications and not a problem. Attentional effects of SNAs have been documented in children (e.g., van Galen and Reitsma, 2008), adults (Dodd, 2011), and synesthetes (Jarick et al., 2009). Finally, SNA-driven attentional biases have recently been generalized into mental arithmetic (Masson and Pesenti, 2014).

The hypothesis that number magnitude biases spatial attention is attractive because this converges with neuroscience facts to explain behavior. For example, healthy adults bisect digit strings made of small, or large numbers to the left or right of center, respectively (Fischer, 2001; Calabria and Rossetti, 2005). Bisection tasks are, in turn, a standard assessment procedure for parietal lobe function (Bonato et al., 2008). Areas in posterior superior parietal cortex (PSPC) are involved in shifting spatial attention and planning saccade targets. PSPC is densely interconnected with the more anteriorly located horizontal aspect of the intraparietal sulcus (hIPS) which processes numerical magnitudes (Nieder and Dehaene, 2009; Eger et al., 2014). Basic mathematical operations may be neurally implemented via functional interactions between hIPS and PSPC and their connection to left perisylvian language areas as well as frontal cortex (Hubbard et al., 2005). Knops et al. (2009) applied machine learning algorithms to the BOLD-activity of PSPC to classify left from right saccades. Crucially, without further training this classifier successfully distinguished mental addition and subtraction, equating addition and subtraction with rightward and leftward saccades, respectively. These results all substantiate the functional role of attention shifts in numerical cognition due to SNAs.

Given these replications, extensions, and conceptual convergence, why did Z&P fail to replicate the original effect? Leaving aside the misleading visualization of their method (see corrigendum, doi: 10.3389/fpsyg.2014.01206), one methodological problem resides with Z&P's decision to obtain evidence for number processing from manual key presses. Participants pressed buttons to indicate digit parity (in Experiments 2 and 5) or magnitude (in Experiments 3 and 6); such responses were not required in the original study. Response preparation was then possible as soon as digits were identified, thus inducing systematic attention shifts for left and right responses that could be either congruent or incongruent with the magnitude-related bias (e.g., Eimer et al., 2005). Although reporting digits after a trial can improve the chance of finding SNAs (e.g., Casarotti et al., 2007), averaging across the manual response conditions dilutes effects of digit presentation on attention allocation. This concern pertains, however, only to 4 of the 6 experiments. Thus, there must be other reasons for why Z&P failed to replicate the original results.

Relatively large samples, as tested by Z&P, do indeed increase statistical sensitivity but also heterogeneity with respect to SNAs: Strength and automaticity of SNAs covary with age and learning history (cf. Wood et al., 2008; Fischer and Shaki, 2014). For example, finger counting is more left-associated in Canada compared to Holland (where the original and replication studies were conducted; Lindemann and Tira, 2011), and this in turn supports SNAs (Fischer, 2008). Similarly, directional reading habits modulate SNAs (Shaki et al., 2009; Shaki and Fischer, 2014). Although not reported in the original study or by Z&P, finger counting preferences and reading habits of participants should be assessed in future studies in light of these recent insights.

Recent evidence and computational theories of selective attention suggest that the salience of a given object in the visual scene relies on the combination of both bottom-up, physical stimulus properties (e.g., luminance or contrast) and its current top-down, strategic relevance for the observer's goals (Fecteau and Munoz, 2006). Hence, context determines the extent of attentional cueing effects, and may likewise determine SNAs. By integrating stimulus-driven conspicuity and strategic relevance the recently proposed idea of priority maps may provide an attractive and coherent framework to investigate the scope and limits of attentional cueing in numerical cognition (Knops et al., 2014). In a similar vein, more evidence is required to elucidate to what extent other well-established attentional mechanisms, such as orienting (benefit due to valid cueing) vs. re-orienting (costs of invalid cueing), differentially contribute to SNAs. For example, in 7–8-year-old children the numerical over- and underestimation during approximate calculation correlates with the costs of invalid attentional cueing (re-orienting) but not with the benefit of valid cueing (orienting) in a Posner paradigm (Knops et al., 2013).

The question whether space is fundamental to number meaning or context-dependent is widely debated (for review see Fischer and Shaki, 2014). We conclude that attentional cueing through number magnitude is indeed subtle and context-dependent but remains a valuable additional tool for investigating conceptual knowledge.

## Conflict of interest statement

The authors declare that the research was conducted in the absence of any commercial or financial relationships that could be construed as a potential conflict of interest.
